# Genome-wide analysis of the maize superoxide dismutase (SOD) gene family reveals important roles in drought and salt responses

**DOI:** 10.1590/1678-4685-GMB-2021-0035

**Published:** 2021-10-01

**Authors:** Jing Liu, Lijuan Xu, Jian Shang, Xiaolin Hu, Haitao Yu, Hongying Wu, Wenben Lv, Yang Zhao

**Affiliations:** 1 Anhui Agricultural University, School of Life Sciences, National Engineering Laboratory of Crop Stress Resistance Breeding, Hefei, China.; 2 Anhui Agricultural University, Maize Engineering Technology Research Center of Anhui Province, School of Life Sciences, Hefei, China.

**Keywords:** Maize, SOD, phylogenetic analysis, expression patterns, abiotic stress

## Abstract

Superoxide dismutase proteins (SODs) are antioxidant enzymes with important roles in abiotic stress responses. The SOD gene family has been systematically analyzed in many plants; however, it is still poorly understood in maize. Here, a bioinformatics analysis of maize *SOD* gene family was conducted by describing gene structure, conserved motifs, phylogenetic relationships, gene duplications, promoter *cis*-elements and GO annotations. In total, 13 *SOD* genes were identified in maize and five members were involved in segmental duplication. Phylogenetic analysis indicated that SODs from maize and other plants comprised two groups, which could be further classified into different subgroups, with most members in the same subgroup having the same subcellular localization. The *ZmSOD* promoters contained 2-10 stress-responsive *cis*-elements with different distributions. Heatmap analysis indicated that *ZmSODs* were expressed in most of the detected tissues and organs. The expression patterns of *ZmSODs* were investigated under drought and salt treatments by qRT-PCR, and most members were responsive to drought or salt stress, especially some *ZmSODs* with significant expression changes were identified, such as *ZmCSD2* and *ZmMSD2*, suggesting the important roles of *ZmSODs* in abiotic stress responses. Our results provide an important basis for further functional study of *ZmSODs* in future study.

## Introduction

Reactive oxygen species (ROS) are inevitable products in the process of cellular metabolism that act as signal molecules to regulate many physiological processes in plants (Gechev *et al.,* 2006). However, abiotic stresses, such as drought, salt and extreme temperature, often induce the production and accumulation of ROS in plant cells (Karuppanapandian *et al.,* 2011), and the presence of excess ROS negatively affects cell growth and even leads to cell death (Mittler, 2002; Lee *et al.,* 2007). Efficient mechanisms have been established to cope with ROS toxicity during the long-term evolution of plants. For example, many studies have shown that some enzymes that remove ROS, such as superoxide dismutase (SOD), peroxidase (POD), catalase (CAT) and glutathione peroxidase (GPX), can protect plants from various abiotic stresses (Mittler *et al.,* 2004; Sugimoto *et al.,* 2014). 

SODs are the first defense of the plant antioxidant system, and play important roles in protecting plants against oxidative stress (Nath *et al.,* 2014; Wang *et al.,* 2017). They mitigate ROS hazards by catalyzing the conversion of superoxide (O^2-^) into hydrogen peroxide (H_2_O_2_) and molecular oxygen (O_2_) under oxidative stress (Gopavajhula *et al.,* 2013), and play significantly roles in protecting the stability of cell membrane and slowing oxidative damage (Karuppanapandian *et al.,* 2011). SODs are very widespread in living organisms. In plants, genome-wide analysis of SOD family genes has been performed in many species, including Arabidopsis (Kliebenstein *et al.,* 1998), rice (Nath *et al.,* 2014), wheat (Jiang *et al.,* 2019), sorghum (Filiz and Tombuloglu, 2015), upland cotton (Wang *et al.,* 2017), and Medicago (Song *et al.,* 2018). These studies have indicated that SODs are encoded by a small gene family; for example, seven *SOD* genes were found in Medicago, and eight members were reported in both of the Arabidopsis and rice genomes. SODs are metalloenzymes, whose proteins require metal cofactors to have catalytic activity (Forman and Fridovich, 1973). Based on the type of metal cofactor, plant SODs can be divided into three groups, iron SODs (FeSODs), manganese SODs (MnSODs), and copper/zinc SODs (Cu/ZnSODs) (Alscher *et al.,* 2002; Fink and Scandalis, 2002). 

Increasing numbers of studies have indicated that *SOD* genes have important roles in response to abiotic stresses (Wang *et al.,* 2004; Pilon *et al.,* 2011; Asensio *et al.,* 2012; Verma *et al.,* 2019). With the development of high-throughput sequencing technology, the expression patterns of SOD family genes in stress responses have been extensively studied. For example, nine *SOD* genes were identified in tomato, and most members showed altered expression under salt and drought stresses according to microarray data analysis (Feng *et al.,* 2016). In Medicago, differential expression was detected for most of the seven *MtSOD* genes under various stress treatments based on microarray analysis and high-throughput sequencing (Song *et al.,* 2018). In foxtail millet, the expression patterns of *SOD* genes were detected under drought, salt, and cold treatments by quantitative real-time PCR (qRT-PCR), and each *SOD* was found to respond to at least one abiotic stress (Wang *et al.,* 2018b). Importantly, the biological functions of some *SOD* genes involved in stress responses have been demonstrated in transgenic plants. For example, Zhang *et al.* (2014) showed that overexpression of a *Tamarix albiflonum SOD* gene, *TaMnSOD*, can improve cotton’s tolerance to drought stress by enhancing root development and the regulation of superoxide scavenging (Zhang *et al.,* 2014). In wheat, overexpression of the *TaSOD2* gene increased salt resistance in transgenic wheat and Arabidopsis plants (Wang *et al.,* 2016).

As an important cereal crop around the world, maize (*Zea mays* L.) has been widely used in genetics and evolution research. However, the growth and yield of maize were seriously affected by various abiotic stresses, and identifying stress-responsive genes and applying them in molecular breeding is one of the effective ways to cope with abiotic stress. Although several *SOD* genes have been identified in maize, systematic analysis of this family has not been reported at whole genome level with the latest genome data, especially for their functional roles in abiotic stress responses (Cannon *et al.,* 1987; Zhu and Scandalios, 1994; Sytykiewicz, 2014). In this study, 13 maize *SOD* genes (*ZmSODs*) were identified in the current genome, and systematic analysis was performed using bioinformatics method. The expression patterns of the 13 genes were also investigated in maize seedlings under drought and salt treatments. The results lay an important foundation for further evolutionary research of plant *SOD* gene family and provide useful information for identification of key *ZmSODs* in response to abiotic stress.

## Material and Methods

### Identification of *ZmSOD* genes in maize

To identify SOD-encoding proteins in the maize genome, Hidden Markov Model (HMM) profiles of the Cu/ZnSOD domain (PF00080) and Fe/MnSOD domains (N-terminal domain, PF00081; C-terminal domain, PF02777) were initially obtained from the Pfam database (http://pfam.xfam.org/) (Finn *et al.,* 2006). Subsequently, we used the HMM profile of each type of SOD proteins as a query to execute a local BLASTP search against the maize genome (v4) (*p*-value = 0.001). All candidate sequences that met the standards were analyzed in the Pfam database to confirm that each sequence contained the related domains. Redundant sequences were removed based on alignments using the ClustalW software (Thompson *et al.,* 1994), and the non-redundant members were used for further analysis. The ExPASy (https://web.expasy.org/protparam/) and WoLF PSORT online tools (https://www.genscript.com/wolf-psort.html) (Horton *et al.,* 2007) were used to predict physico-chemical characteristics and subcellular localizations, respectively. Based on their positions in the genome annotations, the chromosomal distributions of the maize *SOD* genes were displayed from top to bottom on the chromosomes using the MapInspect software. Gene duplication analysis of *ZmSODs* was performed according to previous study (Si *et al.,* 2019).

### Phylogenetic analysis of SOD proteins

To analyze the phylogenetic relationships of SOD proteins among different plants, the full-length amino acid sequences of 37 SOD proteins from maize, Arabidopsis, foxtail millet and rice were used to construct a phylogenetic tree with the MEGA 5.05 (Molecular Evolutionary Genetics Analysis) software. Arabidopsis, foxtail millet and rice SOD sequences were obtained from Joint Genome Institute (http://www.phytozome.net) according to previous studies (Kliebenstein *et al.,* 1998; Nath *et al.,* 2014; Wang *et al.,* 2018b). The phylogenetic tree was built using the neighbor-joining method with 1,000 bootstrap replicates, and the same method was used to construct an unrooted phylogenetic tree of the ZmSOD proteins.

### Conserved motif, gene structure and promoter analysis of *ZmSOD*s

Multiple Em for Motif Elicitation (MEME) (http://meme-suite.org/tools/meme) was used to discover conserved motifs among the 13 maize SOD proteins (Bailey *et al.,* 2009). The motif number was set to 10 and the width of motifs was 6 to 50. The detected motifs were annotated using the Pfam database. Gene Structure Display Server (http://gsds.cbi.pku.edu.cn/index.php) was used to analyze the gene structure by comparing the coding sequence (CDS) with the genomic sequence of each predicted ZmSOD (Hu *et al.,* 2015). To predict putative stress-responsive *cis*-elements in the promoter regions of *ZmSODs*, the 2,000 bp flanking sequences upstream from the transcription start site (ATG) of each *ZmSOD* was obtained from the maize genomic sequence, and these promoter sequences were analyzed using PlantCARE (Lescot *et al.,* 2002).

### Expression patterns of *ZmSOD*s in different tissues and organs

To determine the expression patterns of the *SOD* genes in maize tissues and organs, the publicly available transcriptome data published by Walley *et al.* (2016) for 23 different developmental stages, were downloaded from MaizeGDB (http://www.maizegdb.org/). The fragments per kilobase of transcript per million mapped (FPKM) values were transformed and used to draw a heat map of *ZmSODs* as described in our previous study (Zhao *et al.,* 2019).

### Plant material and stress treatments

The expression levels of the *ZmSODs* were investigated in maize seedlings under abiotic stress conditions. Seeds of the maize inbred line B73 were washed with sterile water three times, and placed in vermiculite for germination until the coleoptile grew to about 2 cm in length. Then, seeds with consistent germination were selected, washed with water, and placed on plastic tanks containing Hoagland’s nutrient solution in a plant growth chamber at 28 °C/23 °C (day/night) with a 16-h light/8-h dark photoperiod. At the three-leaf stage, the seedlings were used for drought and salt treatments to explore the possible functional roles of the *ZmSODs* in response to abiotic stress. Hoagland’s nutrient solution containing 20% (m/v) PEG-6000 or 200 mM NaCl was used for drought and salt treatment, respectively. At 0, 3, 6, 12, and 24 h after treatment, the third leaf of each seedling was harvested, and immediately frozen in liquid nitrogen, and stored at -80°C for RNA extraction.

### RNA isolation and quantitative real-time PCR (qRT-PCR) analysis

Total RNA was extracted from the seedling samples using AG RNAex Pro Reagent (Accurate Biology, China). RNA quality and concentration were assessed by 1% agarose gel electrophoresis and P200+ Series Micro Volume Spectrophotometers (Pultton, USA), respectively. First-strand cDNA was generated from 1 μg of total RNA using HiScript® III RT SuperMix for qPCR (+ gDNA wiper) (Vazyme, China) according to the manufacturer’s instructions. Gene-specific primers were designed using the Primer3Plus online tool, and the NCBI database was used to verify the specificity of the primers (Table S1). qRT-PCR reactions were carried out as described in our previous study (Zhao *et al.,* 2019). The maize *GAPDH* gene (accession number: NM_001111943.1) was utilized as an internal control for normalizing expression levels. Three biological and three technical repeats were performed for each gene.

## Results

### Identification of SOD proteins in maize

Using the local BLASTP program, a total of 13 non-redundant SOD proteins were obtained with predicted Cu/ZnSOD or Fe/MnSOD domains after confirmation with the Pfam database. The number of SOD proteins in the maize genome was significantly higher than in Arabidopsis and rice. Based on their phylogenetic relationships, chromosomal distributions and metal cofactors, the 13 *SOD* genes were named *ZmCSD1*-*ZmCSD6*, *ZmFSD1*-*ZmFSD5*, *ZmMSD1* and *ZmMSD2*. According to the physico-chemical characteristics predicted by the Expasy tool, we found that the protein lengths, molecular weights (MWs), and isoelectric points (pI) of the ZmSOD members had large ranges. The protein lengths of the ZmSODs ranged from 152 to 386 aa, and the molecular weights of the ZmSODs varied from 15.07 to 42.87 kDa. The isoelectric points of the ZmSODs ranged from 5.33 to 8.84. According to the subcellular localization predictions, the highest number of members was localized in mitochondria, including ZmFSD1, ZmFSD2, ZmFSD3, ZmFSD5, ZmMSD1 and ZmMSD2, while only three proteins (ZmCSD3, ZmCSD4 and ZmFSD4) were localized in chloroplasts. In addition, four proteins, including ZmCSD1, ZmCSD2, ZmCSD5, and ZmCSD6, were localized in cytoplasm (Table 1).

**Table 1 -:** Sequence characteristics of the 13 *SOD* genes identified in maize.

Gene name	Sequence ID	Protein length (aa)	MW (kDa)	pI	Subcellular predicted
*ZmCSD1*	Zm00001d028232_T004	163	16.82	6.23	Cytoplasm
*ZmCSD2*	Zm00001d029170_T003	152	15.17	5.83	Cytoplasm
*ZmCSD3*	Zm00001d031908_T001	206	20.90	5.45	Chloroplast
*ZmCSD4*	Zm00001d002611_T002	308	32.11	5.33	Chloroplast
*ZmCSD5*	Zm00001d022505_T001	233	24.92	5.91	Cytoplasm
*ZmCSD6*	Zm00001d047479_T003	152	15.07	5.64	Cytoplasm
*ZmFSD1*	Zm00001d014632_T001	154	17.80	6.58	Mitochondrion
*ZmFSD2*	Zm00001d036135_T003	284	32.43	8.84	Mitochondrion
*ZmFSD3*	Zm00001d045384_T003	201	23.02	7.14	Mitochondrion
*ZmFSD4*	Zm00001d045538_T001	386	42.87	5.63	Chloroplast
*ZmFSD5*	Zm00001d025106_T003	186	21.50	6.65	Mitochondrion
*ZmMSD1*	Zm00001d037859_T001	213	22.84	6.71	Mitochondrion
*ZmMSD2*	Zm00001d009990_T001	243	26.44	6.71	Mitochondrion

### Phylogenetic relationships and gene structure

**Figure 1 -:**
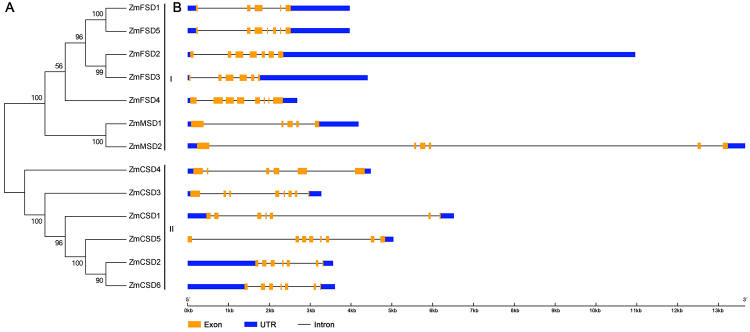
Phylogenetic relationships and gene structures of maize SOD proteins. A. Unrooted tree of the 13 ZmSODs. The tree was constructed with 1,000 bootstrap replicates by the neighbor-joining method using the MEGA5.05 software. B. Exon-intron structures of the *ZmSOD*s.

The full-length ZmSOD sequences were aligned and used to construct an unrooted phylogenetic tree to analyze their phylogenetic relationships. The result indicated that the 13 ZmSODs could be divided into two groups (I-II) with high bootstrap value support, indicating their conserved phylogenetic relationships (Figure 1A). To further support the phylogenetic relationships of the ZmSODs, gene structure analysis was performed for the 13 *ZmSODs* using GSDS online tool (Figure 1B). We found that intron numbers in the genomic sequences of the *ZmSODs* ranged from 4 to 7. Three *ZmSODs* (*ZmCSD3*, *ZmCSD5* and *ZmFSD4*), contained seven introns, while *ZmFSD1* and *ZmMSD1* contained four introns. According to the phylogenetic tree and gene structure analysis, we found that the gene pair *ZmCSD2*-*ZmCSD6* exhibited a highly similar exon-intron organization pattern, suggesting their close relationship.

**Figure 2 -:**
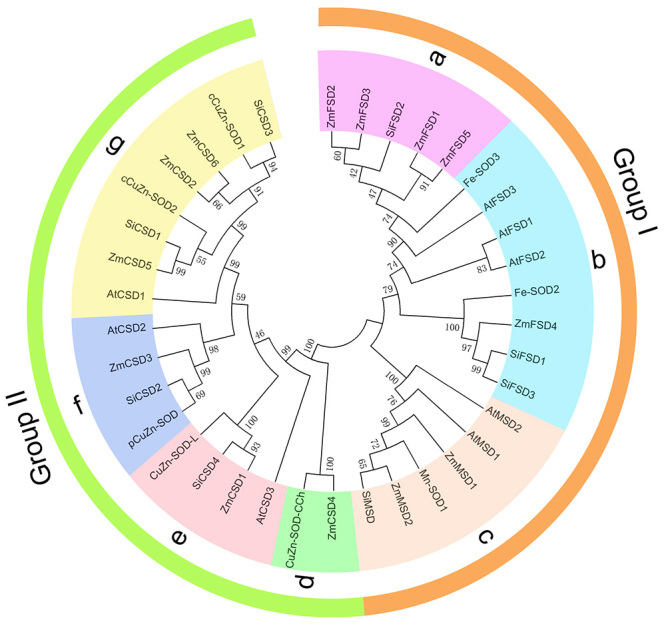
Phylogenetic relationships of 37 SOD proteins from different plant species.

To further investigate the phylogenetic relationships of SOD proteins in dicot and monocot plants, a phylogenetic tree was constructed based on an alignments of 37 full-length protein sequences, including 13 sequences from maize, 8 from foxtail millet (SiCSD1, SiCSD2, SiCSD3, SiCSD4, SiFSD1, SiFSD2, SiFSD3 and SiMSD), 8 from rice (cCuZn-SOD1, cCuZn-SOD2, CuZn-SOD-L, pCuZn-SOD, CuZn-SOD-CCh, Fe-SOD3, Fe-SOD2 and Mn-SOD1), and 8 from Arabidopsis (AtCSD1, AtCSD2, AtCSD3, AtFSD1, AtFSD2, AtFSD3, AtMSD1 and AtMSD2). According to the phylogenetic tree, the 37 SOD proteins could be divided into two groups: group I (Fe/MnSODs) and group II (Cu/ZnSODs), which was consistent with the types of domains they contained (Figure 2). Group I contained 19 SOD proteins, which could be further divided into three subgroups (a-c). In group II, 18 SOD proteins were divided into four subgroups (d-g). We found that most SOD proteins clustered in the same subgroups shared the same predicted subcellular localization (Table 1). For example, ZmFSD1, ZmFSD2, ZmFSD3, ZmFSD5 and other plants’ mitochondrial FeSODs formed subgroup a. ZmCSD2, ZmCSD5 and ZmCSD6 were contained in subgroup g, and these ZmSODs and other plant SOD proteins were predicted to be localized in the cytoplasm. In each subgroup, we found that the ZmSODs exhibited closer relationships with foxtail millet or rice members than those of Arabidopsis. We also constructed a maximum likelihood (ML) tree with the same SOD protein sequences using MEGA 5.05 software, and the results were largely consistent with the phylogenetic relationships in the NJ tree (Figure S1), which further supported the reconstruction of the NJ tree.

### Conserved motifs, chromosomal distributions and gene duplications

**Figure 3 -:**
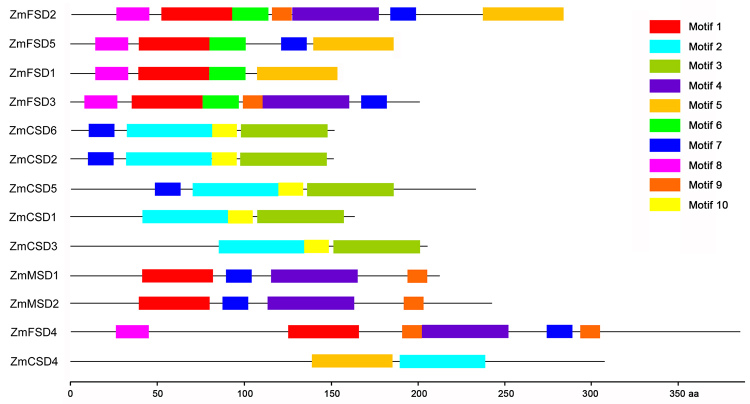
Conserved motif analysis of ZmSOD proteins.

**Figure 4 -:**
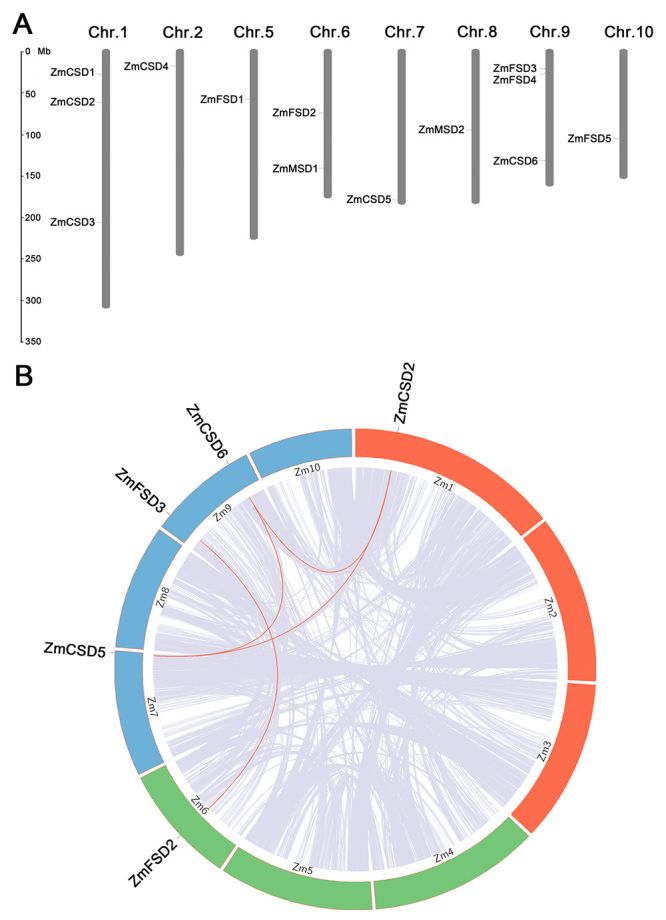
Chromosomal locations and syntenic analysis of the *ZmSOD* genes. A. Chromosomal locations of the 13 *ZmSODs*. B. Synteny and gene duplication analysis among *ZmSODs* in the maize genome.

MEME was used to investigate the conserved motifs among ZmSODs, and 10 motifs were identified (Table S2). For the Cu/ZnSOD proteins, motifs 2 and 3 encoding the Cu/ZnSOD domain (PF00080) were detected in each of the six ZmCSDs except ZmCSD4 (Figure 3). Motif 1 encoding a Fe/MnSOD domain (N-terminal domain, PF00081) was found in each of the Fe/MnSOD proteins, while Motif 5 encodes a Fe/MnSOD domain (C-terminal domain, PF02777), was detected in ZmFSD1, ZmFSD2 and ZmFSD5. Notably, ZmSODs in the same phylogenetic group tended to have similar motif distribution patterns, which further supported the phylogenetic classification. The chromosomal locations of the *ZmSOD*s were obtained from the maize genome database. As shown in Figure 4A, eight of the 10 chromosomes harbored *ZmSOD*s; no *ZmSOD* genes were found on chromosomes 3 or 4. Most of the genes were distributed on chromosomes 1, 6 and 9, while chromosomes 2, 5, 7, 8 and 10 each contained only one *SOD* gene. Gene duplications, including tandem and segmental duplications, were investigated to explore the potential expansion mechanism of *ZmSOD* family. According to the syntenic analysis, five genes (*ZmCSD2*, *ZmCSD5*, *ZmCSD6*, *ZmFSD2* and *ZmFSD3*) were involved in the segmental duplication, and no tandem duplications were detected in *ZmSOD* gene family (Figure 4B).

### Promoter analysis of *ZmSOD*s

**Figure 5 -:**
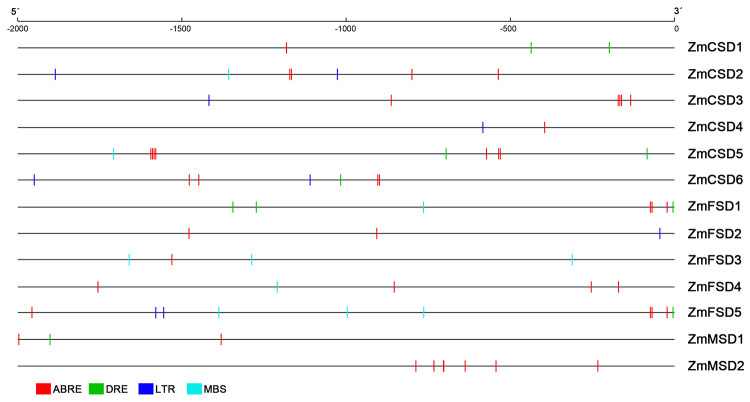
*Cis*-elements in the promoter regions of the *ZmSOD* genes. Four types of putative stress-responsive *cis*-elements, including ABRE, DRE, LTR and MBS, were shown with different colors.

Increasing evidence indicates that *SOD* genes play important roles in responses to abiotic stresses. To explore the possible regulatory mechanisms of *ZmSODs* involved in stress responses, the putative stress-responsive *cis*-elements were investigated in the promoter sequences of the *ZmSODs*. Four *cis*-elements, including the abscisic acid responsiveness element (ABRE), dehydration-responsive element (DRE), MYB binding site involved in drought-inducibility (MBS) and low temperature-responsive element (LTR), were detected in this study. The number of detected *cis*-elements in the 13 promoter regions ranged from 2 to 10 (Figure 5). Each of the *ZmFSD5* and *ZmCSD5* promoters had 10 *cis*-elements, respectively, while *ZmCSD4* had the least number (2). The distributions of *cis*-elements in the *ZmSOD* promoters showed significant differences, which might suggest the different roles or regulatory mechanisms of *ZmSOD*s in responses to abiotic stresses.

### Gene Ontology (GO) enrichment analysis of *ZmSOD*s

**Figure 6 -:**
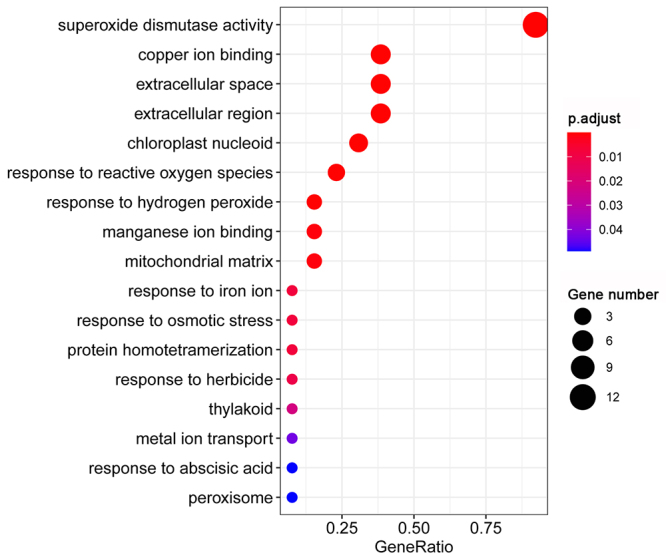
Gene Ontology (GO) enrichment analysis of the *ZmSOD* genes.

To explore the possible functional roles of the *ZmSODs*, GO terms for these genes were annotated using the clusterProfiler R package (Yu *et al.,* 2012), and divided into three categories, including cellular component, molecular function, and biological process (Figure 6). The results indicated that the *ZmSODs* were significantly (adjusted *p*-value < 0.05) enriched in 17 GO terms. Six GO terms, including extracellular space (GO:0005615), chloroplast nucleoid (GO:0042644), extracellular region (GO:0005576), mitochondrial matrix (GO:0005759), thylakoid (GO:0009579) and peroxisome (GO:0005777), were enriched in the cellular component category. Three GO terms, including superoxide dismutase activity (GO:0004784), copper ion binding (GO:0005507), and manganese ion binding (GO:0030145), were enriched in the molecular function category. Eight GO terms, including response to reactive oxygen species (GO:0000302), response to hydrogen peroxide (GO:0042542), response to iron ion (GO:0010039), response to osmotic stress (GO:0006970), protein homotetramerization (GO:0051289), response to herbicide (GO:0009635), metal ion transport (GO:0030001) and response to abscisic acid (GO:0009737), were enriched in the biological process category. We noted that the highest number of *ZmSODs* (12) was enriched in the “superoxide dismutase activity” molecular function term, three genes were enriched in the “response to reactive oxygen species” biological process term, and one gene was enriched in the “response to osmotic stress” biological process terms. These results suggested that *ZmSODs* have significant roles in the responses to abiotic stress.

### Expression patterns of *ZmSOD*s at different developmental stages

**Figure 7 -:**
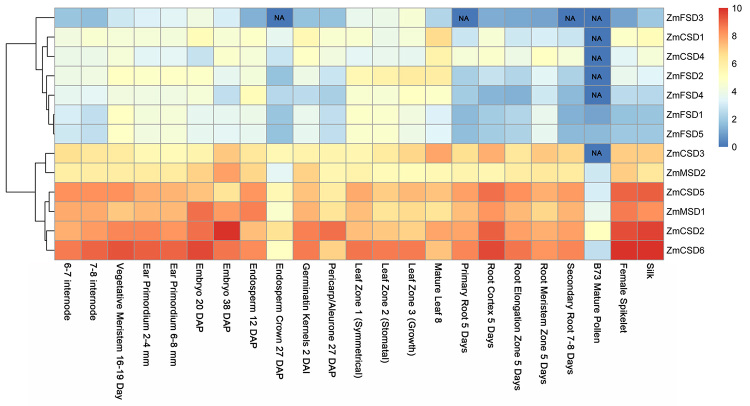
Expression pattern analysis of the *ZmSOD* genes in different tissues and organs using transcript data. Expression patterns of the 13 *ZmSODs* were investigated in 23 different tissues and organs using publicly transcriptome data. FPKM showed “NA” (not available) was replaced by FPKM = 0, and all FPKM values were transformed into log_2_ (FPKM+1) to create the heatmap.

To explore the possible functions of the *ZmSOD*s, the expression patterns of the 13 *ZmSOD*s were analyzed in 23 different tissues and organs using publicly available transcript data (Walley *et al.,* 2016). All the 13 *ZmSODs* showed detectable expression levels in most of the 23 tissues and developmental stages (Figure 7) with different expression patterns. According to their expression levels, the 13 *ZmSODs* could be divided into two groups. The first group included 7 members (*ZmFSD3*, *ZmCSD1*, *ZmCSD4*, *ZmFSD2*, *ZmFSD4*, *ZmFSD1* and *ZmFSD5*) with low expression, while the second group exhibited relatively higher expression, including *ZmCSD3*, *ZmMSD2*, *ZmCSD5*, *ZmMSD1*, *ZmCSD2* and *ZmCSD6*. We noted that *ZmMSD* genes had high expression levels in all stages except B73 mature pollen while *ZmFSD* genes had low expression levels in most stages. In addition, some *SOD* genes showed similar expression patterns that reflected their close relationships, especially for two pairs of genes (*ZmCSD2* and *ZmCSD6*, and *ZmFSD1* and *ZmFSD5*), which might suggest their similar functions in plant growth and development.

### Expression analysis of *ZmSOD*s under drought and salt treatments

**Figure 8 -:**
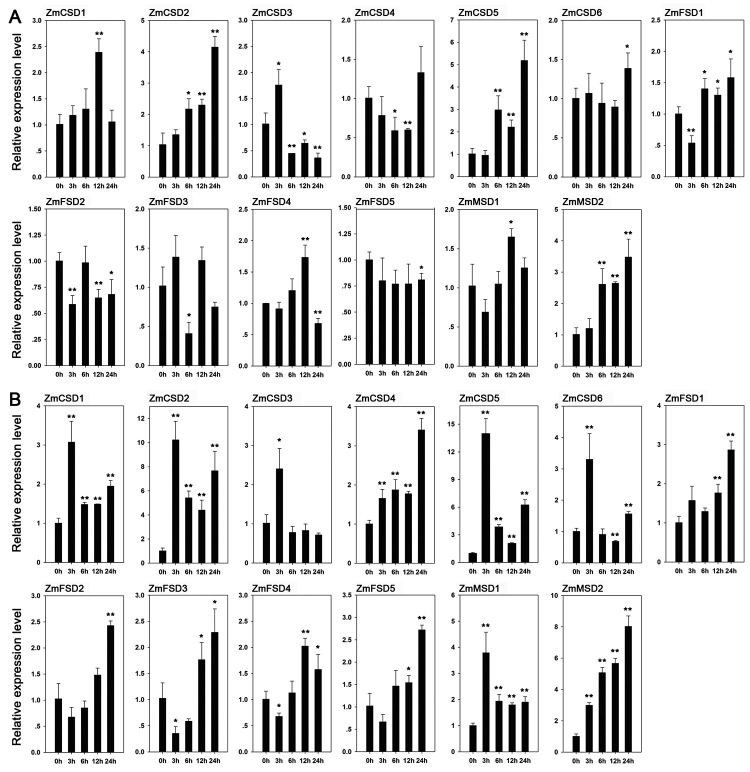
Expression pattern analysis of *ZmSOD* genes under drought (A) and salt (B) treatments by qRT-PCR. The X-axis is the time course of treatment, and seedlings were sampled at 0 (CK), 3, 6, 12 and 24 h after drought or salt treatment, respectively. The Y-axis shows the relative expression levels.

The expression levels of the *ZmSOD*s were investigated under drought and NaCl treatments using qRT-PCR to understand their possible roles in responses to abiotic stresses. Under drought stress, expression levels of *ZmCSD2*, *ZmCSD5* and *ZmMSD2* were significantly up-regulated with large fold changes at the 6, 12 and 24 h, respectively, while *ZmFSD2* was significantly down-regulated at three of the four time points (Figure 8A). Notably, some *ZmSODs* exhibited significant differences across the four time points. For example, *ZmCSD3* expression was significantly up-regulated at 3 h, but significantly down-regulated expression was observed at 6, 12 and 24 h. *ZmFSD4* expression was significantly up-regulated at 12 h, but significantly down-regulated was shown at 24 h after drought treatment. In addition, some *ZmSODs* exhibited significant up- or down-regulated expression only at particular time points, such as *ZmCSD1* and *ZmFSD5*. Under salt stress, we found that expression levels of *ZmCSD1*, *ZmCSD2*, *ZmCSD4*, *ZmCSD5*, *ZmMSD1* and *ZmMSD2* were significantly up-regulated at all of the four time points (Figure 8B). As observed under drought stress, significant expression differences were observed for some *ZmSODs* across the four time points, for example, *ZmFSD3* exhibited significantly up-regulated expression at 12 and 24 h, and significantly down-regulated expression was observed at 3 h. In addition, significantly up-regulated expression was only observed at particular time points for *ZmCSD3* and *ZmFSD2*. Under salt treatment, we found that the expression levels of most *ZmSODs* exhibited larger fold changes than that observed under drought stress. These findings suggested the important roles of *ZmSODs* in responses to drought or salt stress, but may have different regulatory mechanisms.

## Discussion

Environmental stresses, such as drought, heat and salinity, have serious effects on plant growth and development. Studies have indicated that ROS accumulation can causes oxidative stress because the equilibrium of oxidative reactions is disrupted by abiotic stresses (Apel and Hirt, 2004; Quan *et al.,* 2008). Toxic ROS can greatly harm the survival of plants, such as through inactivation of enzymes and membrane lipids (Apel and Hirt, 2004). SOD is one of the antioxidant enzymes that have been demonstrated to have important roles in protecting plant cells from oxidative damage (Fink and Scandalis, 2002). *SOD* family genes have been studied in several plant species, and identification of the maize *SOD* genes and determination of their functional roles in responses to drought and salt stresses will provide excellent gene resources for resistance breeding to various abiotic stresses.

Studies indicated that the number of *SOD* family genes has a large difference among different plant species. For example, 8 *SOD* genes were identified in both of rice and foxtail millet, while 26 members were identified from the whole genome of wheat. A total of 13 *SOD* genes were identified in the current maize genome, and further divided into two major types. Although there are large differences in the genome sizes of different plant species, the number of *SOD* family genes is not proportional to the genome size. It is believed that gene duplications, including tandem and segmental duplications, have important roles in the expansion of plant gene families (Cannon *et al.,* 2004). The different numbers of *SOD* family genes should be mainly attributable to the ratio of gene duplications. Our study indicated that five *ZmSODs* were involved in the segmental duplication, which suggests that segmental duplication plays an important role in the expansion of maize *SOD* gene family. 

Phylogenetic analysis indicated that the 37 SOD proteins from four species were divided into two groups, termed group I (Fe/MnSODs) and group II (Cu/ZnSODs). As observed in other studies, FeSODs and MnSODs from different plants were clustered together in group I with high bootstrap value support, suggesting that these genes may share common ancestral genes (Wang *et al.,* 2017; Song *et al.,* 2018). SODs from monocots (maize, foxtail millet, and rice) and dicots (Arabidopsis) tended to cluster separately, which was consistent with the evolutionary relationships of these species. The findings also suggested the independent evolution of plant *SOD* genes after the divergence of monocots and dicots. Interestingly, some maize SODs exhibited close phylogenetic relationships with their orthologs from other species than their paralog proteins, suggesting that the ortholog pairs may have originated from a common ancestor and diverged after the divergence of the grass genome. Our study was consistent with previous studies that *SOD* genes clustered in one phylogenetic branch tended to have the same subcellular localization (Song *et al.,* 2018; Wang *et al.,* 2018b), further supporting the phylogenetic reconstruction and the conserved evolution of plant *SOD* genes. 

*Cis*-elements in gene promoters are important for transcriptional regulation (Hernandez-Garcia and Finer, 2014). Therefore, stress-responsive *cis*-elements, including ABRE, DRE, MBS and LTR, were investigated in the promoters of the 13 *ZmSODs*. Previous studies indicated these stress-responsive *cis*-elements have important roles in regulating abiotic stress responses. For example, expression of *RD29A* was induced by drought, salt, ABA and low-temperature, both of DRE and ABRE elements were found in its promoter (Shinozaki and Yamaguchi-Shinozaki, 2000; Narusaka *et al.,* 2003). In maize, mutant analysis indicated that MBS element in *ZmSO* promoter was important for ABA- and drought-induced expression (Xu *et al.,* 2019). In addition, LTR was shown to be involved in regulation of cold responsive, and it was also found in some drought or salt responsive gene promoters (Wang *et al.,* 2018a; Xie *et al.,* 2019). We found that at least one type of the four stress-responsive *cis*-elements was detected in each of the *ZmSODs*, suggesting the important roles of the *ZmSODs* in abiotic stress responses. However, different distribution patterns were found among the 13 *ZmSODs*, even for the gene pair *ZmCSD2*-*ZmCSD6*. These findings may indicate different regulatory mechanisms of the *ZmSODs* in response to abiotic stress.

Plant growth and development is frequently threatened by various environmental stresses (Zhu, 2002; Gong *et al.,* 2020; Lozano-Juste *et al.,* 2020). At present, the expression patterns of *SOD* genes in response to abiotic stress have been investigated in many species, and the functional studies of some genes involved in stress response have also been demonstrated by biological experiments (Zhang *et al.,* 2014; Wang *et al.,* 2016). According to the expression levels of *ZmSODs* under stress treatments, we found that most of the *ZmSODs* showed significant response to drought or salt stress treatment. Meanwhile, the significant expression differences among the 13 *ZmSODs* were also found. Some members exhibited similar expression patterns under different stresses. For example, significantly up-regulated expression of *ZmCSD2* and *ZmMSD2* was detected under both of drought and salt treatments, suggesting their conserved functional roles of these genes in response to abiotic stresses. However, different expression patterns between drought and salt treatments were also found. For example, *ZmCSD4* exhibited significantly down-regulated expression at 6 and 12 h under drought stress, but significantly up-regulated expression was detected across the four time points under salt stress. In foxtail millet, the expression of *SiMSD* was significantly induced by drought and salt stresses, respectively (Wang *et al.,*2018b). *ZmMSD2* had a close phylogenetic relationship with *SiMSD*, and also exhibited significantly up-regulated expression under drought and salt stresses, which may indicate their conserved functions in stress responses. Importantly, we should note that some *ZmSODs* were up-regulated with a large fold changes under drought or salt treatment, such as *ZmCSD2*, *ZmCSD5* and *ZmMSD2*. Our study provided an important foundation for the selection of important functional genes and application in stress resistance breeding in maize.

## Conclusions

In this study, 13 maize *SOD* genes were identified using the BLASTP program and systematic bioinformatics analysis was performed for these members. The 13 *ZmSODs* were distributed on 8 of the 10 maize chromosomes, and five members were involved in segmental duplication, suggesting that segmental duplication plays an important role in the expansion of maize *SOD* gene family. SOD proteins from maize and three other plants members were divided into two groups (Fe/MnSODs and Cu/ZnSODs) according to phylogenetic analysis, which can be further classified into different subgroups. At least one of the four detect stress-responsive *cis*-elements was identified in each of the *ZmSODs*. Transcriptome data analysis showed that *ZmSODs* were expressed in most of the detected tissues and organs. Furthermore, qRT-PCR analysis indicated that most of the *ZmSODs* were responsive to drought or salt stress treatments, especially some genes with significant expression changes were identified. Our results lay an important foundation for further identifying important members and investigating the molecular functions of *ZmSODs* involved in abiotic stress responses.

## Supplementary Material

The following online material is available for this article:

Figure S1 - Maximum likelihood phylogenetic tree of 37 SOD proteins from different plant species. The Maximum Likelihood (ML) tree was constructed.

Table S1 - Gene-specific primers used for qRT-PCR in this study.

Table S2 - Conserved motif sequences of maize SOD proteins.
