# Supplementary material for: Genome-wide analysis of the maize superoxide dismutase (SOD) gene family reveals important roles in drought and salt responses
Source: Genet Mol Biol. 2021 Oct 1;44(3):e20210035. doi: 10.1590/1678-4685-GMB-2021-0035 (PMC8493800; doi:10.1590/1678-4685-GMB-2021-0035)
Supplement: Figure S1 - [file 1415-4757-GMB-44-3-e20210035-s1.pdf]

Genetics and Molecular Biology

https://doi.org/10.1590/1678-4685-GMB-2021-0035

Supplementary Material to "Genome-wide analysis of the maize superoxide dismutase (SOD) gene family reveals important roles in drought and salt responses"

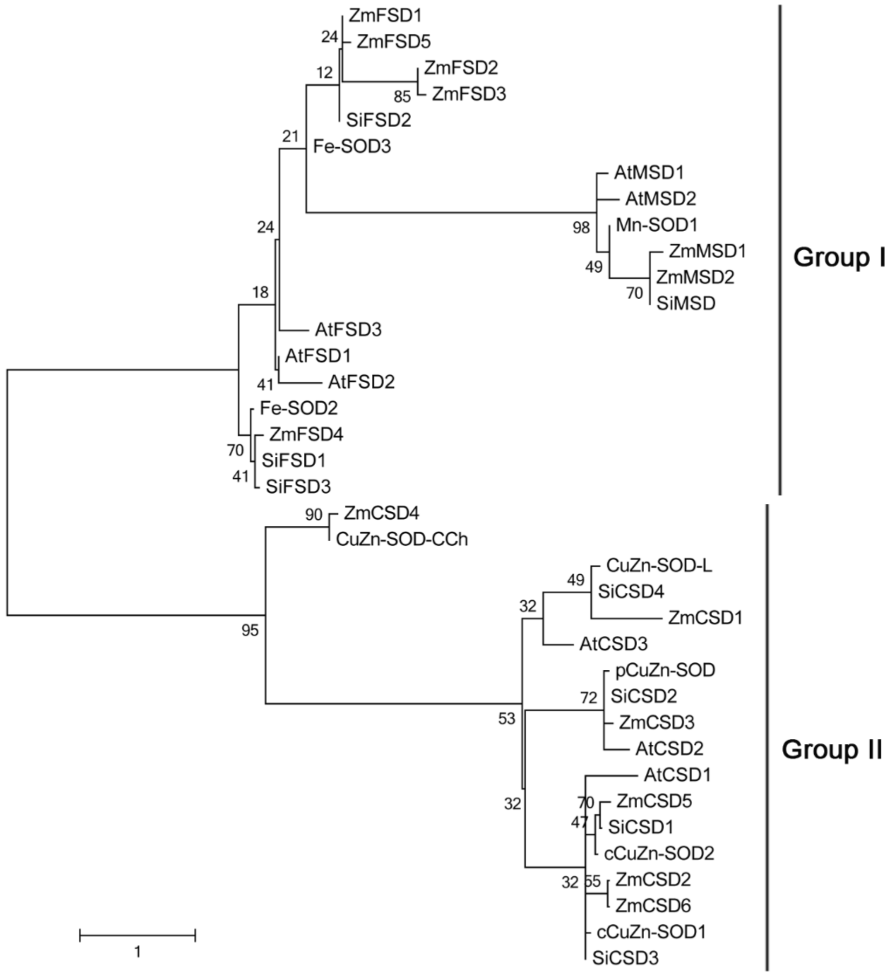

Figure S1 - Maximum likelihood phylogenetic tree of 37 SOD proteins from different plant species. The Maximum Likelihood (ML) tree was constructed using MEGA 5.05 software by alignments of the full-length sequences of the 37 SOD proteins. Bootstrap values from 1,000 replicates were shown at each branch.
