# Supplementary material for: Genome-wide analysis of the maize superoxide dismutase (SOD) gene family reveals important roles in drought and salt responses
Source: Genet Mol Biol. 2021 Oct 1;44(3):e20210035. doi: 10.1590/1678-4685-GMB-2021-0035 (PMC8493800; doi:10.1590/1678-4685-GMB-2021-0035)
Supplement: Table S1 - [file 1415-4757-GMB-44-3-e20210035-s2.pdf]

Genetics and Molecular Biology

https://doi.org/10.1590/1678-4685-GMB-2021-0035

Supplementary Material to "Genome-wide analysis of the maize superoxide dismutase (SOD) gene family reveals important roles in drought and salt responses"

Table S1 - Gene-specific primers used for qRT-PCR in this study.

Gene names	Primer 5´-->3´
GAPDH	F: ATCAACGGCTTCGGAAGGAT<br>R: CCGTGGACGGTGTCGTACTT
ZmCSD1	F: TACAGTCGGTGAGGAAATTG<br>R: CAGAATGCACTCACAAAAGG
ZmCSD2	F: GGCTGAACTGAAACAAATGG<br>R: CAGCATTTTAGCCAACAGTC
ZmCSD3	F: GTTATTGGGACAGTGACCTT<br>R: CGATTTGGATATGTGGCAAC
ZmCSD4	F: CTTCATCAAACAGGTCGAGA<br>R: CTCTAGCCAACTCCATGTTT
ZmCSD5	F: TCCATGATCAGAGAGCACTA<br>R: ACCTCCAGTAGTGTCTTCTT
ZmCSD6	F: GCTGATCTGTGATCATCGTA<br>R: GCTGACTGCATATCAAAACC
ZmFSD1	F: CACAGCACAGCACGCTTA<br>R: GGCTCACCAAGGTTCACAAA
ZmFSD2	F: GAAACTGCGCTCGAAAATAC<br>R: GTTCCAGAGCATCCAGTTTA
ZmFSD3	F: ACGAAGGACACGAAGAATAG<br>R: TATCACCCGATTTCCGATTC
ZmFSD4	F: CAAAGACTCCAAATGCCATC<br>R: CAGAAACATAGTCAGCCCTT
ZmFSD5	F: GTTGGGTTTGGCTTGTCTT<br>R: GTAAGCGTGAATGTCACCAA
ZmMSD1	F: GACTTTGTTGTCGTGCTAAG<br>R: ACATTTTCGTACACCTCTCC
ZmMSD2	F: CAAACCTTAACCTCCCCTTC<br>R: ACTCGACTCTCGTAGGTG
