# Supplementary material for: Genome-wide analysis of the maize superoxide dismutase (SOD) gene family reveals important roles in drought and salt responses
Source: Genet Mol Biol. 2021 Oct 1;44(3):e20210035. doi: 10.1590/1678-4685-GMB-2021-0035 (PMC8493800; doi:10.1590/1678-4685-GMB-2021-0035)
Supplement: Table S2 - [file 1415-4757-GMB-44-3-e20210035-s3.pdf]

Genetics and Molecular Biology

https://doi.org/10.1590/1678-4685-GMB-2021-0035

Supplementary Material to "Genome-wide analysis of the maize superoxide dismutase (SOD) gene family reveals important roles in drought and salt responses"

Table S2 - Conserved motif sequences of maize SOD proteins.

Motifs	Width (aa)	Sequences
1	41	YKLDALEPYMSKRTVELHWGKHHQDYVDGLNKQLATSPLYG
2	49	SVSGLKPGLHGFHIHALGDTTNGCMSTGPHYNPANKEHGAPEDENRHAG
3	50	TDSQIPLTGPNSIIGRAVVVHADPDDLGKGGHELSKSTGNAGGRVACGII
4	50	SMQPGGGGPPEGKLLQAIDKDFGSFEALVKEFIAEALALLGSGWVWLALK
5	47	YYLDYKDDKLTYVTNFMDHLVSWHTVTLRMMRAESFVNLGEPNILEA
6	21	YTLEDLIKEAYNNGNPLPEYN
7	15	VVHVKGAIFFTAEGD
8	19	KKIQRQGGLSRRFSKVVYY
9	11	AQVWNHHFYWZ
10	15	DLGNVTAGADGVANV
